# Nomogram for medication nonadherence risk prediction in post-valve surgery patients: a retrospective study

**DOI:** 10.3389/fmed.2026.1675779

**Published:** 2026-03-19

**Authors:** Wenjuan Ye, Hanxiang Ma, Ke Yang, Yan Xu, Xiaoyan Wang, Cuicui Peng, Lancai Guo

**Affiliations:** Department of Cardiovascular Surgery, The First Affiliated Hospital of Anhui Medical University, Hefei, China

**Keywords:** medication adherence scale, medication noadherence, nomogram, post-valvular surgery, prediction model

## Abstract

**Purpose:**

To develop and internally validate a nomogram for individualized prediction of post-valvular surgery medication nonadherence risk.

**Materials and methods:**

We developed a prediction model in 244 post-valvular surgery patients enrolled between March 2025 and July 2025. Medication adherence was assessed using the Adherence to Refills and Medications Scale (ARMS). Among the 244 included patients, 112 were classified as nonadherent (ARMS score >16). Predictor selection was performed using least absolute shrinkage and selection operator (LASSO) regression, followed by multivariable logistic regression for nomogram construction. With 112 outcome events and five predictors in the final model, the events-per-variable ratio was 22.4, supporting sample size adequacy for exploratory prediction model development. Model performance was evaluated by the concordance index (C-index), area under the receiver operating characteristic curve (AUC), calibration, and decision curve analysis (DCA). Internal validation was performed using 1,000 bootstrap resamples in accordance with TRIPOD-oriented reporting principles.

**Results:**

The final nomogram incorporated five key predictors: use of warfarin, children accompany, dosing frequency daily, education level, and distance to hospital. The model demonstrated excellent discrimination, with a C-index of 0.839 (95% CI: 0.808–0.870) in the training cohort, and maintained strong predictive performance during internal validation (C-index = 0.833). Calibration plots indicated good agreement between predicted and observed probabilities. Decision curve analysis showed that the nonadherence nomogram was clinically useful when the threshold was between 12 and 68%. The AUC was found to be 0.817 [95% CI = 0.784–0.845] in the training set.

**Conclusion:**

This validated nomogram incorporating warfarin use, children accompany, dosing frequency daily, education level, and distance to hospital provides a practical tool for individualized prediction of medication nonadherence risk in post-valvular surgery patients.

## Introduction

Valvular Heart Disease (VHD) is a heart disease that causes a single or multiple heart valve function or structural abnormality due to various causes, resulting in valve stenosis/or insufficiency. Heart valve replacement surgery is the most fundamental treatment method for heart valve diseases, which greatly improves the patient’s heart function and saves the patient’s life (1). Medication nonadherence is defined as the act of discontinuing or stopping treatment for the prescribed duration (2). Nonadherence with prescribed drug regimens is a pervasive medical problem. Multiple variables affecting physicians and patients contribute to nonadherence, which negatively affects treatment outcomes. For post-valvular surgery patients, adherence to long-term therapy in patients is associated with relieving symptoms, decreasing disease flares, and controlling disease progress (3). In addition, the consequences of poor adherence to long-term therapies are poor health outcomes and increased health care cost (4, 5).

Standardized pharmacotherapy following valvular heart surgery is crucial for optimizing surgical outcomes, preventing complications, and improving long-term survival and quality of life. However, post-discharge medication adherence remains suboptimal due to limited access to systematic, long-term therapeutic guidance. Furthermore, socioeconomic factors (e.g., financial constraints, health literacy, social support), self-efficacy, disease severity, and adverse drug reactions contribute to poor medication management—including inappropriate dose adjustments, inadequate INR monitoring, and premature discontinuation—ultimately compromising clinical prognosis (4). Poor medication adherence in chronic diseases is a global challenge with significant clinical and public health implications (5, 6). Medication nonadherence in diseases is a multifactorial issue, driven by socioeconomic, clinical, therapeutic, and patient-specific barriers, including: Socioeconomic factors (e.g., social support, marital status, family cohesion); Disease-related factors (e.g., disease severity, functional status, insurance coverage); Treatment-related factors (e.g., dosing complexity, polypharmacy, adverse effects); Patient-related factors (e.g., demographics, socioeconomic status, healthcare access, education level, and distance to hospital) (6). Considering so many associated risk factors, accurate prediction adherence tools and early intervention may be the most effective actions toward unsatisfactory adherence (7). The Adherence to Refills and Medications Scale (ARMS) is a suitable adherence measurement and has been developed and identified to assess the effective adherence to medicine (8). ARMS can be used to identify variables related to nonadherence. Nowadays, a nomogram as a visually predictive tool calculates the risk of outcomes for individuals. This tool provides valuable guidance for clinical decision-making. Based on ARMS, a predictive nomogram might make a difference for post-valvular surgery patients who might present medication nonadherence. According to this model, clinicians achieve an individual probability of post-valvular surgery patients at risk of medication nonadherence and make interventions.

This study aimed to develop a simple yet valid prediction tool, based on ARMS adherence assessment, to identify post-valvular surgery patients at risk of medication nonadherence using readily available baseline characteristics.

## Patients and methods

### Patients

Ethical approval for this study was granted by the Ethics Committee of the First Affiliated Hospital of Anhui Medical University (Approval No. PJ2022-12-34). Consecutive patients who underwent heart valve surgery and were successfully discharged between March 2025 and July 2025 were recruited from the First Affiliated Hospital of Anhui Medical University. All participants provided written informed consent prior to study enrollment. Exclusion criteria comprised illiteracy, severe cognitive impairment, or significant physical disability precluding participation. Each enrolled patient completed adherence-assessment questionnaires and participated in a structured 5-min interview with a specialist pharmacy assistant. Patient demographic, disease-related, and treatment characteristics were abstracted from electronic medical records.

### Adherence assessment

Medication adherence was assessed using the ARMS (8). Based on the ARMS scoring protocol, there were 12 items and two dimensions in ARMS: medication compliance (7 items) and continuation compliance (5 items). Likert 4-point scale was used for each item, 1 point = never (Never), 2 points = sometimes (Sometimes), 3 points = often (Often), 4 points = always (Always). Participants with a total score ≤ 16 were categorized into the adherence group, while those scoring >16 were categorized into the non-adherence group. The scale demonstrated good psychometric properties: internal consistency was acceptable, content validity was high, and test–retest reliability was excellent (Cronbach’s *α* = 0.814).

### Statistical analysis

Categorical variables, including demographic, disease-related, and treatment-related characteristics, are presented as numbers and percentages. All analyses were performed using R software (version 4.5.1; R Foundation for Statistical Computing). The “rms” and “rmda” packages were used for modeling and evaluation. All statistical tests were two-sided, and *p* < 0.05 was considered statistically significant. Nineteen candidate variables were initially considered. To reduce dimensionality and limit overfitting, we first applied least absolute shrinkage and selection operator (LASSO) regression; predictors with non-zero coefficients were then entered into a multivariable logistic regression model to construct the nomogram (9). Because the study was exploratory and prediction-oriented, all predictors retained by LASSO were preserved in the final multivariable model for nomogram development, even if individual Wald *p*-values were not statistically significant. Results are reported as regression coefficients, odds ratios (ORs), 95% confidence intervals (CIs), and p-values. The final model contained five predictors and 112 nonadherence events, corresponding to an events-per-variable ratio of 22.4, which is generally considered adequate for stable exploratory model development. Model reporting was revised with reference to TRIPOD recommendations to improve transparency.

Calibration curves were generated to assess agreement between predicted and observed risk (10–12). Discrimination was quantified using Harrell’s concordance index (C-index) and the area under the receiver operating characteristic curve (AUC). Internal validation was performed with 1,000 bootstrap resamples to estimate a bias-corrected C-index (13). Decision curve analysis was used to evaluate the potential clinical utility of the nomogram by quantifying net benefit across a range of threshold probabilities (14, 15). Given the single-center retrospective design, no external or temporal validation cohort was available; therefore, the current model should be interpreted as an internally validated exploratory prediction tool.

## Results

### Patients’ characteristics

**Figure 1:**
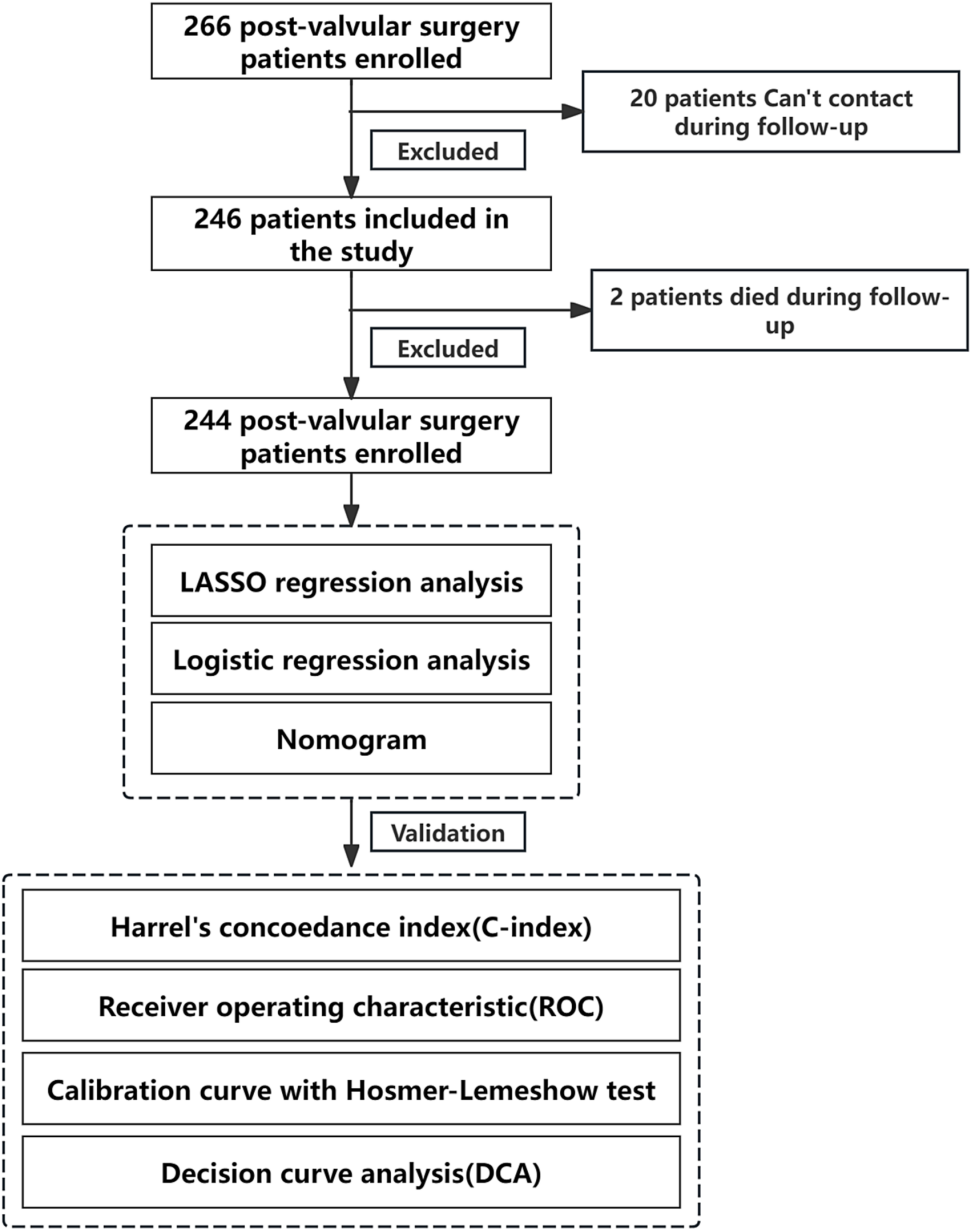
Flowchart of this study.

A total of 266 patients who underwent valvular surgery were enrolled from March to July 2025. Twenty patients were lost to follow-up, and two died during follow-up, resulting in 244 patients who completed the final questionnaire. Based on ARMS scores, patients were categorized into adherence and nonadherence groups (119 male, 125 female; mean age, 63.57 ± 14.89 years; range, 46–77 years). Data on demographic, disease, and treatment characteristics were collected and are presented in Table 1 for both groups (Figure 1).

**Table 1:** Baseline characteristics between demographic and clinical characteristics of adherent and nonadherent groups.

Demographic characteristics	*N* (%)		
	Adherence (*n* = 132)	Nonadherence (*n* = 112)	Total (*n* = 244)
Age (years)			
<55	31 (23.48)	32 (28.57)	63 (25.82)
≥55	101 (76.52)	80 (71.43)	181 (74.18)
Sex			
Female	65 (49.24)	60 (53.57)	125 (51.23)
Male	67 (50.76)	52 (46.43)	119 (48.77)
Marital status			
Married	122 (92.42)	103 (91.96)	225 (92.21)
Other marital statuses	10 (7.58)	9 (8.04)	19 (7.79)
Education level			
Primary (0–9 years)	55 (41.67)	30 (26.79)	85 (34.84)
Secondary (9–12 years)	42 (31.82)	40 (35.71)	82 (33.60)
Higher (>12 years)	35 (26.51)	42 (37.50)	77 (31.56)
Employment			
Employed	80 (60.61)	44 (39.29)	124 (50.82)
Unemployed	52 (39.39)	68 (60.71)	120 (49.18)
Working strength			
Less activity (office, and so on)	52 (39.39)	46 (41.07)	98 (40.16)
Light-to-moderate activity (installers and so on)	47 (35.61)	43 (38.39)	90 (36.89)
Moderate or heavy activity (agriculture and so on)	33 (25.00)	23 (20.54)	56 (22.95)
Monthly per capita income (yuan)			
<1,000	5 (3.79)	6 (5.36)	11 (4.51)
1,000–9,999	102 (77.27)	86 (76.79)	188 (77.05)
10,000–19,999	12 (9.09)	11 (9.82)	23 (9.43)
>20,000	13 (9.85)	9 (8.03)	22 (9.01)
Type of medical insurance			
Rural cooperative medical care	56 (42.42)	52 (46.43)	108 (44.26)
Urban medical insurance	45 (34.09)	41 (36.61)	86 (35.25)
Other insurance	31 (23.49)	19 (16.96)	50 (20.49)
Distance to hospital (km)			
≥50	64 (48.48)	42 (37.50)	106 (43.44)
<50	68 (51.52)	70 (62.50)	138 (56.56)
Beyond annual household income			
Yes	62 (46.97)	51 (45.54)	113 (46.31)
No	70 (53.03)	61 (54.46)	131 (53.69)
Disease duration (years)			
0–1	29 (21.97)	21 (18.75)	50 (20.49)
1–5	83 (62.88)	63 (56.25)	146 (59.84)
≥5	20 (15.15)	28 (25.00)	48 (19.67)
Comorbidities			
0	50 (37.88)	60 (53.57)	110 (45.08)
1–2	52 (39.39)	34 (30.36)	86 (35.25)
≥3	30 (22.73)	18 (16.07)	48 (19.67)
Types of pills prescribed daily			
1–2	27 (20.45)	8 (7.14)	35 (14.34)
3	24 (18.18)	18 (16.07)	42 (17.21)
4–5	62 (46.97)	32 (28.57)	94 (38.52)
≥6	19 (14.40)	54 (48.22)	73 (29.92)
Children accompanied			
Yes	96 (72.73)	62 (55.36)	158 (64.75)
No	36 (27.27)	50 (44.64)	86 (35.25)
Use of Warfarin			
Yes	90 (68.18)	72 (64.29)	162 (66.39)
No	42 (31.82)	40 (35.71)	82 (33.61)
Dosing frequency daily			
<once	9 (6.82)	2 (1.79)	11 (4.51)
once	13 (9.85)	12 (10.71)	25 (10.25)
≥twice	110 (83.33)	98 (87.50)	208 (85.24)
Types of side effects			
0	31 (23.48)	20 (17.86)	51 (20.90)
1	56 (42.42)	36 (32.14)	92 (37.70)
2	36 (27.27)	40 (35.71)	76 (31.15)
≥3	9 (6.83)	16 (14.29)	25 (10.25)
Consultation frequency yearly			
1–4	45 (34.09)	36 (32.14)	81 (33.20)
5–10	56 (42.42)	55 (49.11)	111 (45.49)
≥10	31 (23.49)	21 (18.75)	52 (21.31)
Hospitalization frequency yearly			
Never	84 (63.64)	65 (58.04)	149 (61.07)
Once	33 (25.00)	37 (33.04)	70 (28.69)
≥Twice	15 (11.36)	10 (8.92)	25 (10.24)

### Feature selection

**Figure 2:**
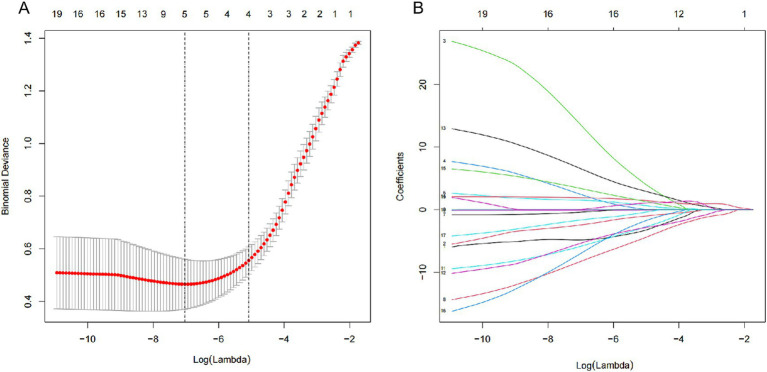
Demographic and clinical feature selection using the LASSO binary logistic regression model. **(A)** Optimal parameter (lambda) selection in the LASSO model used fivefold cross-validation via minimum criteria. The partial likelihood deviance (binomial deviance) curve was plotted versus log (lambda). Dotted vertical lines were drawn at the optimal values by using the minimum criteria and the 1 SE of the minimum criteria (the 1-SE criteria). **(B)** LASSO coefficient profiles of the 19 features. A coefficient profile plot was produced against the log(lambda) sequence. Vertical line was drawn at the value selected using fivefold cross-validation, where optimal lambda resulted in five features with nonzero coefficients. LASSO, least absolute shrinkage and selection operator; SE, standard error.

Among demographic, disease, and treatment features, 19 features were initially considered. Using LASSO regression on data from 244 patients in the cohort (Figures 2A,B), five features emerged as potential predictors with nonzero coefficients. These included warfarin use, children accompany, daily dosing frequency, education level, and distance to the hospital (Table 2).

**Table 2:** Prediction factors for medication nonadherence.

Intercept and variable	Prediction model		
	*β*	Odds ratio (95% CI)	*p*-value
Intercept	−2.2399	0.106 (0,072–0.153)	<0.001
Children accompanied	−0.0602	0.942 (0.637–1.391)	0.762
Use of Warfarin	2.5794	4.184 (2.348–7.539)	0.007
Dosing frequency daily	1.4312	1.899 (1.190–3.028)	0.632
Education level	−1.5375	0.215 (0.011–1.303)	<0.001
Distance	1.0090	2.743 (1.501–5.027)	0.001

β is the regression coefficient. CI, confidence interval.

### Development and validation of prediction model

**Figure 3:**
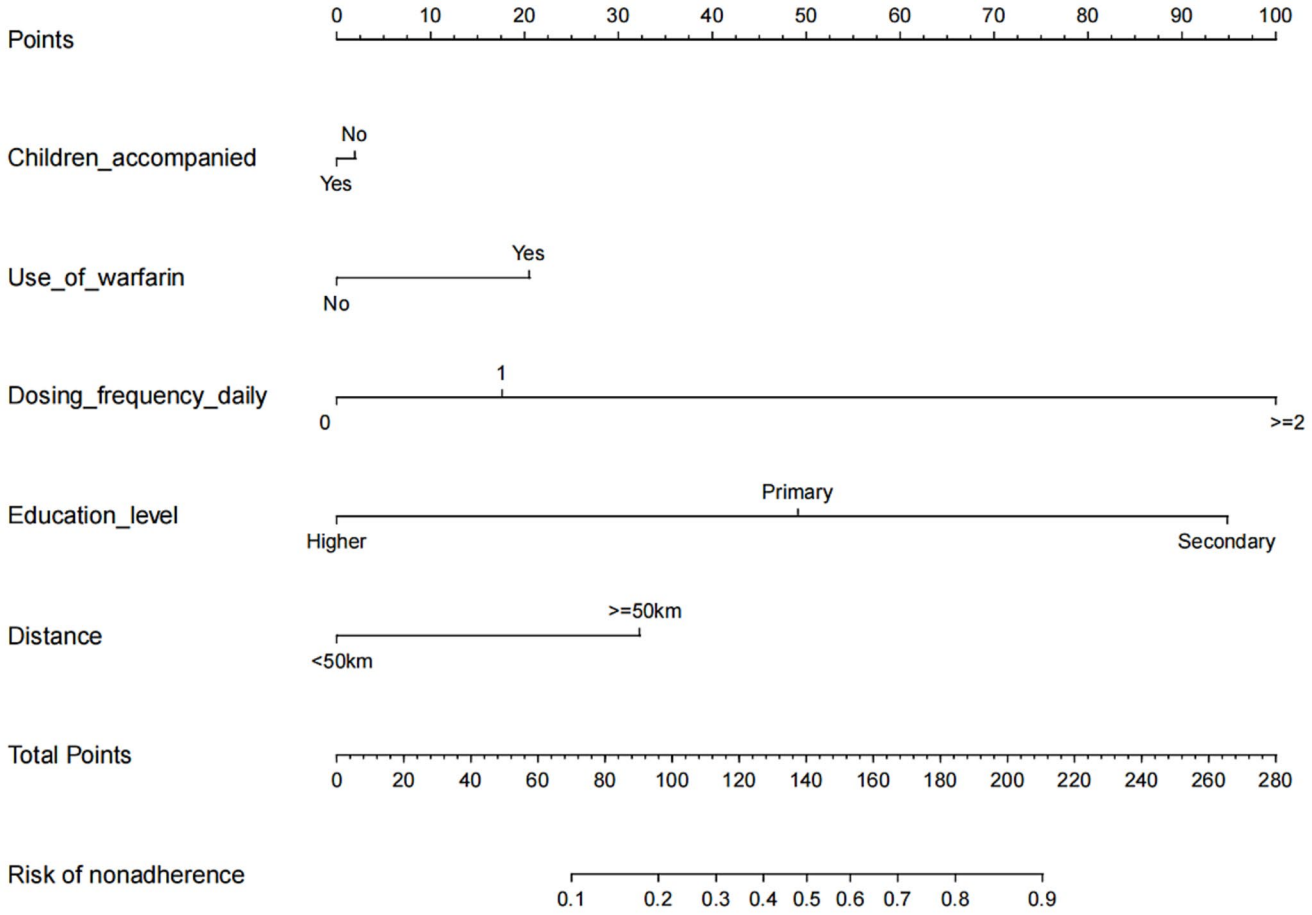
Developed medication nonadherence nomogram. The medication nonadherence nomogram was developed in the cohort, with the children accompanied, the use of warfarin, the dosing frequency daily, education level, and the distance to hospital incorporated.

**Figure 4:**
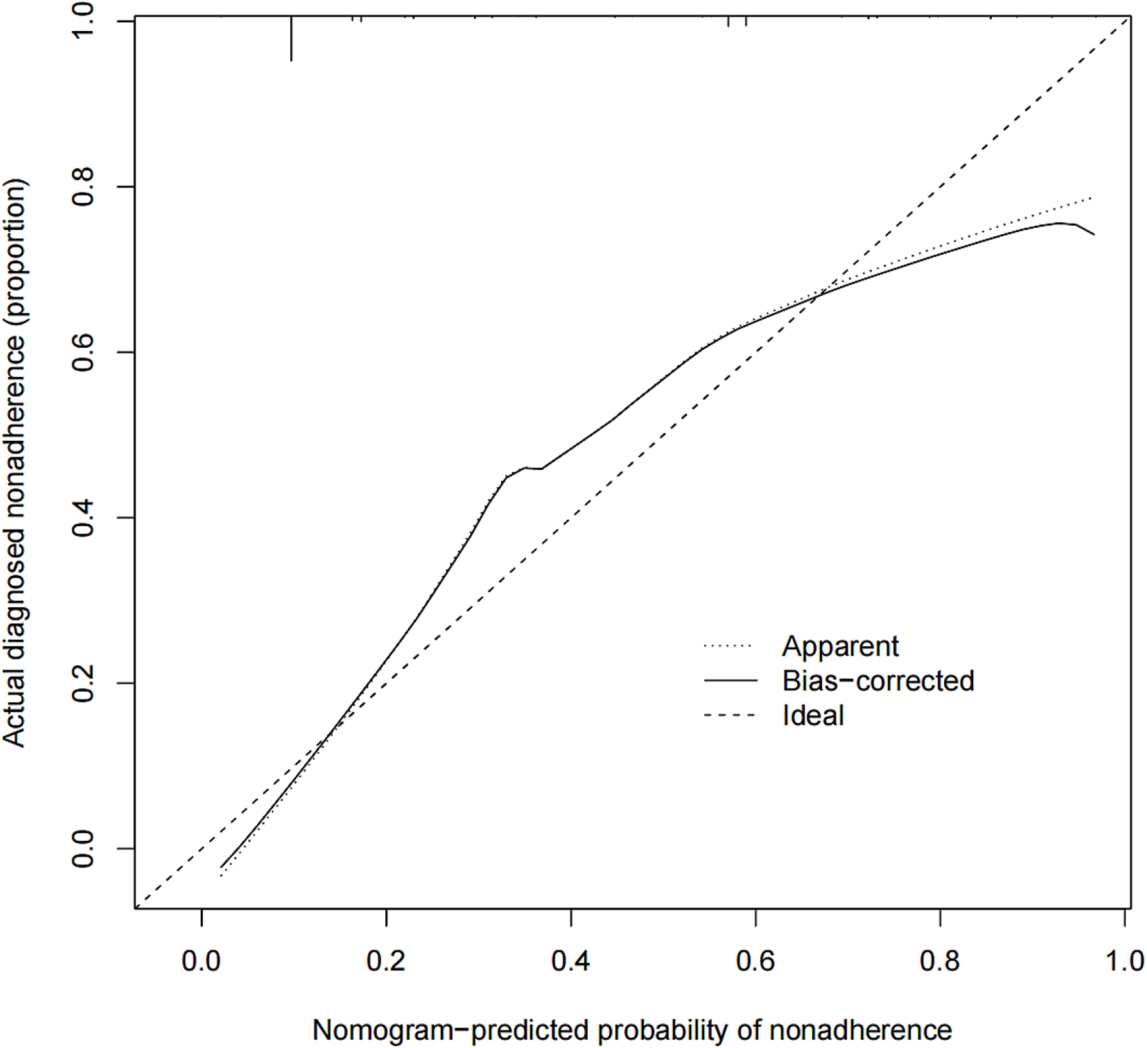
Calibration curves of the nonadherence nomogram prediction in the cohort. The *x*-axis represents the predicted medication nonadherence risk. The *y*-axis represents the actual diagnosed nonadherence. The diagonal dotted line represents a perfect prediction by an ideal model. The solid line represents the performance of the nomogram, of which a closer fit to the diagonal dotted line represents a better prediction.

**Figure 5:**
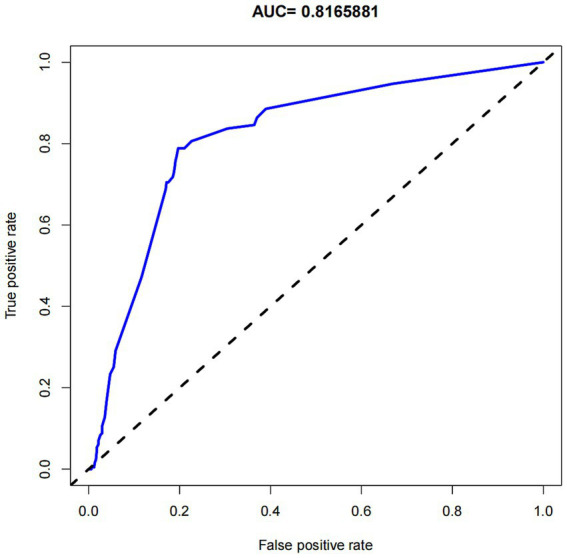
The area under the receiver operating characteristic curve (AUC) for the predictive model.

We developed a nomogram to predict medication nonadherence risk in post-valvular surgery patients. This model incorporated five independent predictors: use of warfarin, children accompany, dosing frequency daily, education level, and distance to the hospital (Figure 3). The calibration curve demonstrated good agreement between predicted and observed nonadherence risk in the cohort (Figure 4). The nomogram exhibited excellent discrimination, with a C-index of 0.839 (95% CI: 0.808–0.870). Bootstrap validation confirmed this discriminative performance (C-index: 0.833). The AUC value of this model is 0.817 (Figure 5). Collectively, the nomogram demonstrated robust predictive capability.

### Clinical use

**Figure 6:**
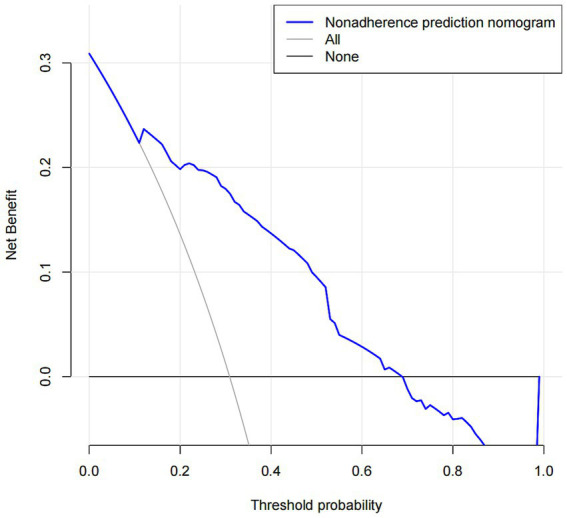
Decision curve analysis for the nonadherence nomogram. The *y*-axis measures the net benefit. The dotted line represents the medication nonadherence risk nomogram. The thin solid line represents the assumption that all patients are nonadherent to medication. Thin thick solid line represents the assumption that no patients are nonadherent to medication. The decision curve showed that if the threshold probability of a patient and a doctor is >12 and <68%, respectively, using this nonadherence nomogram in the current study to predict medication nonadherence risk adds more benefit than the intervention-all-patients scheme or the intervention-none scheme.

Figure 6 displays the decision curve analysis (DCA) of the medication nonadherence prediction nomogram. The analysis revealed that when the threshold probability for clinical intervention ranged from 12 to 68%, implementing this nomogram to stratify nonadherence risk yielded superior net benefit compared to both treat-all and treat-none strategies. Throughout this clinically significant probability spectrum, the nomogram demonstrated comparable net benefit to alternative approaches, with substantial overlap observed between the decision curves.

## Discussion

Valvular Heart Disease (VHD), characterized by high rates of disability and mortality, significantly impairs patient quality of life (16). Driven by evolving lifestyles, improved living standards, accelerated societal pace, and cumulative stress factors, the prevalence of VHD continues to rise, with an increasing incidence among younger populations (17). While surgical intervention or transcatheter prosthetic valve replacement offers substantial clinical improvement for VHD patients, a critical challenge persists post-discharge: many patients lack access to systematic, long-term medication management guidance. Consequently, medication non-adherence manifesting as self-adjusted dosing or premature discontinuation is frequently observed. This non-adherence stems from multifaceted barriers including socioeconomic constraints, educational limitations, inadequate social support, low self-efficacy, disease complexity, and adverse drug effects (18). Such non-adherence poses significant risks, potentially leading to thromboembolic events (19), valve obstruction (due to thrombosis or pannus formation) (20), and hemorrhagic complications associated with antithrombotic therapy (21). These adverse outcomes can profoundly compromise long-term prognosis. Therefore, strict adherence to evidence-based pharmacological regimens following valvular intervention is a cornerstone for optimizing surgical outcomes, preventing valve-related morbidity, and enhancing long-term survival and quality of life (22, 23).

Currently, nomograms are widely utilized as prognostic tools in oncology and medicine. These tools leverage intuitive digital platforms, enhanced accuracy, and improved interpretability of prognostic estimates to facilitate superior clinical decision-making (24). To our knowledge, this represents the first application of a nomogram to predict medication nonadherence risk in post-valvular surgery patients. We developed and validated a novel nomogram-based prediction tool for medication nonadherence risk in post-valvular surgery patients, utilizing five readily accessible clinical and sociodemographic variables: warfarin use, cohabitation status with children, daily dosing frequency, education level, and distance to hospital. This clinically applicable nomogram facilitates individualized risk stratification for medication nonadherence in this population. Internal validation demonstrated favorable discrimination and calibration. Validation in the cohort yielded a high C-index, supporting the model’s robust performance and potential for broader clinical utility. This nomogram suggested that using no warfarin, children accompany, higher education, shorter distance to hospital, and less dosing frequency daily may be the key individual factors that determine medication nonadherence risk for post-valvular surgery patients.

Following heart valve surgery, patients require long-term anticoagulation therapy involving regular international normalized ratio (INR) monitoring and dietary modifications (25, 26). However, as the duration post-discharge extends and patient’s transition from a primarily patient role to reintegrated family and societal roles, adherence to anticoagulation therapy often declines among both patients and their families. This waning vigilance manifests as delayed or missed medication doses, skipped INR checks, deviations from prescribed dietary guidelines, and even treatment discontinuation. Such non-adherence not only compromises surgical outcomes but also significantly increases the risk of severe thromboembolic or hemorrhagic complications, including stroke and intracranial hemorrhage (27). Studies indicate that while medication adherence is generally favorable following heart valve replacement, patients often exhibit suboptimal lifestyle management and inadequate international normalized ratio (INR) monitoring. This discrepancy may stem from insufficient long-term, structured health education and medication guidance, both during hospitalization and after discharge, despite patients’ general awareness of anticoagulation therapy’s importance. These findings underscore the critical need for sustained patient care beyond the acute postoperative phase. Leveraging advancements in internet-based technologies, continuous patient management can be facilitated through platforms such as smart healthcare devices, text messaging (28, 29). Such interventions promote active patient participation, thereby enhancing engagement and initiative in anticoagulation self-management, ultimately improving long-term treatment adherence. The results of this study show that patients with high levels of education have better adherence in the post-valvular surgery patients, which is consistent with other research results (30, 31). The possible reason is that patients with high education have better understanding of disease knowledge and related medications, have stronger ability to obtain medical knowledge from multiple aspects and channels, and have better self-control ability to transform knowledge into behavior (32). The results of this study show that the better medication adherence with anticoagulant treatment of patients who live with their family, the reason may be related to physical inconvenience, fear of exercise, limitations in relevant knowledge, economic pressure, rehabilitation and social obstruction after heart valve surgery. Patients with high family support get more care, higher emotional value, stronger family happiness, and better medical behavior (33). Family plays an important role in medication adherence of patients receiving life-long anticoagulant treatment, especially in domestic life after discharge. Family functioning is recognized as a critical determinant of health outcomes in patients with cardiovascular disease (34). Defined as the processes by which families communicate, fulfill roles, manage stress, and maintain cohesion, family functioning significantly influences long-term self-management and treatment adherence. Evidence demonstrates that patients reporting higher levels of family functioning-characterized by substantial support and care-exhibit superior medication adherence behaviors (35). Due to the great trauma of heart valve surgery, coupled with postoperative pain, long-term medication treatment, rehabilitation of primary diseases, personality, family, and social support, some patients have anxiety and depression, or serious depression and clinically related anxiety, which seriously affects the patient’s physical and mental health and leads to postoperative related complications. Family and children accompanying each other are conducive to sootheing the patient’s mood, promoting a positive psychological state, improving personal coping ability, and in order to prevent and reduce postoperative complications, promote prognosis and improve quality of life, patients will actively acquire disease knowledge and adopt active coping strategies to face the disease, thereby improving medication adherence after valve surgery (36).

In addition, this study found that the frequency of daily medications can also affect patients’ medication nonadherence after heart valve surgery. High pill burden associated with certain therapies have been linked to poor adherence. Reducing pill burden through the use of once-daily formulations has proven valuable in improving adherence to evidence-based therapies (37). Consequently, interventions such as medication reminders and regular follow-up target adherence must be tailored to the particular illness-related demands experienced by the patients (4, 22). Disease therapy and demographic factors are sometimes difficult to change, but clinicians and pharmacists play a crucial role in solving drug problems, especially after heart valve surgery. Longer travel distances to healthcare facilities are associated with potentially worse medication adherence. Patients following heart valve surgery require regular clinical follow-up; however, those in the early postoperative period or elderly patients often necessitate caregiver accompaniment. Furthermore, transportation barriers and other logistical challenges contribute to increased disease burden, which may negatively impact adherence to medication regimens in this population.

The patients with better adherence to medication showed better outcomes compared to those with poor adherence, which demonstrated that developing nonadherence risk prediction tools might improve patient outcomes with individualized risk prediction and interventions. This study developed a valid nonadherence risk prediction tool, which assisted clinicians with early identification of patients at high risk of nonadherence to medication. In addition, it may serve as a guide for the optimal selection of VHD patients in clinical research. Accurate prognostic assessment of medication nonadherence risk enables clinicians to identify high-risk patients promptly, facilitating timely interventions. This approach can prevent unnecessary testing in low-risk scenarios and avoid treatment delays or discontinuities when therapeutic net benefit is likely high. However, predicting nonadherence at the individual patient level remains challenging. Addressing suboptimal adherence effectively may require tailored measurement strategies combined with multifaceted interventions. Furthermore, the paucity of studies investigating determinants of long-term medication persistence, coupled with the scarcity of research on predictors of adherence to short-term regimens, highlights critical areas warranting future investigation.

### Limitations

This study has several limitations. First, the retrospective design inherently precludes the establishment of causal relationships (38). Furthermore, the analysis was constrained by the availability of data within the medical records, limiting our ability to account for potentially important confounding factors (39, 40). Consequently, prospective studies are warranted to validate the observed associations and establish causality. Second, model development and validation were performed solely on an internal cohort. While this facilitated rigorous model optimization within a controlled setting, it inherently limits the model’s generalizability to external populations. Therefore, external validation using independent cohorts from diverse institutions is essential to confirm the model’s broader applicability. Third, the single-center nature of this study introduces potential selection and information biases inherent to data derived from a single institution. Thus, future multicenter, prospective cohort studies are imperative to mitigate these biases and provide more robust evidence.

## Conclusion

A novel nomogram was developed to accurately stratify medication nonadherence risk in post-valvular surgery patients. Providing individual risk estimates, it facilitates tailored lifestyle monitoring and medical intervention strategies by clinicians and patients. While requiring external validation, subsequent research must evaluate if personalized approaches informed by this tool effectively lower nonadherence rates and enhance clinical outcomes.

## Data Availability

The original contributions presented in the study are included in the article/supplementary material, further inquiries can be directed to the corresponding author/s.
